# Editorial: Cytokine Release Syndrome in COVID-19: Mechanisms and Management

**DOI:** 10.3389/fphar.2022.965714

**Published:** 2022-08-09

**Authors:** Tao Hu

**Affiliations:** Evergreen Therapeutics Inc., Bethesda, MD, United States

**Keywords:** cytokine release syndrome, SARS-CoV-2, COVID-19, 17α-hydroxyprogesterone caproate, selective serotonin reuptake inhibitor

Cytokine release syndrome is known to be a leading cause of morbidity and mortality in coronavirus disease 2019 (COVID-19) patients (Que et al., 2022). Severe acute respiratory syndrome coronavirus 2 (SARS-CoV-2) infection triggers activation of immune system and excessive production of pro-inflammatory cytokines, leading to tissue damage, disseminated intravascular coagulation, acute respiratory distress syndrome, multiorgan failure, and death (Mangalmurti and Hunter, 2020). Favorable management of cytokine release syndrome by suppressing hyper-inflammatory responses has been expected to exhibit therapeutic effects in moderate to severe COVID-19 patients. The monoclonal antibody tocilizumab, for instance, exerts potent anti-inflammatory effects by blocking interleukin (IL)-6 receptor. In severe COVID-19 patients, the use of tocilizumab was shown to be significantly associated with a reduced risk of invasive mechanical ventilation and death (Abidi et al., 2022). In June 2021, an emergency use authorization was issued by the US. Food and Drug Administration (FDA) to tocilizumab for the treatment of certain hospitalized patients with COVID-19. Despite significant progresses made to calm the cytokine storm, the underlying mechanisms by which SARS-CoV-2 infection induces cytokine release syndrome still need to be fully elucidated, novel and effective therapeutic agents to mitigate hyper-inflammatory responses in COVID-19 patients remain highly warranted.

The current thematic issue aims to provide the readers with research evidences and better understandings of the pathogenesis of COVID-19, with emphases on the development of cytokine release syndrome associated with SARS-CoV-2 infection and driving forces. Additionally, this issue also presents research efforts to develop potential therapies targeting hyper-inflammatory responses and excessive production of cytokines in COVID-19. To these ends, 4 articles were eventually accepted to be published in this Research Topic, including 1 report of meta-analysis, 1 review and 2 research papers.

The meta-analysis by Hu et al. from China demonstrated the strong association between elevated circulating cytokines and COVID-19 severity and mortality. The authors screened 13,468 studies from PubMed, Embase, Web of Science, and other databases from December 2019 to June 2021, a total of 77 articles with 13,986 patients were ultimately included in this study. Their findings showed that circulating levels of IL-2R, IL-10, IL-1β, IL-4, IL-8, IL-17, tumor necrosis factor (TNF)-α, and particularly IL-6, were elevated in severe and non-surviving COVID-19 patients, when compared with mild patients. The serum IL-6 level in the non-surviving group was also much higher than that in the surviving group. Importantly, the alternations of cytokine levels in COVID-19 showed gender and ethnicity differences. Of particular note, the levels of IL-6, IL-2R, IL-10 and TNF-α in male patients were significantly higher than that in female patients, consistent with the more severe symptoms and higher mortality observed in males (Takahashi et al., 2020).

Among the key cytokines and chemokines increased in COVID-19, the review by Cesta et al. from Italy and the US. highlighted IL-8 as a biomarker and prognostic factor in modulating the hyper-inflammatory responses in acute respiratory distress syndrome associated with SARS-CoV-2 infection. IL-8 showed remarkable increases in the blood, the bronchoalveolar lavage fluid, and the lungs of COVID-19 patients, and exhibited a notable compartmentalized response within the lungs, consistent with its well-established role in the recruitment of neutrophils to the lungs during acute pulmonary inflammation (Ronit et al., 2021). Given its active involvement in mediating inflammatory responses and disease pathogenesis in COVID-19, modulation of IL-8 signaling is expected to exhibit therapeutic potential. Currently, two agents targeting IL-8 signaling have entered clinical trials to test their therapeutic efficacies in the treatment of hospitalized patients with severe COVID-19, including human monoclonal antibody HuMax-IL-8 (BMS986253) and reparixin, an allosteric inhibitor of IL-8.

In the pursuit of potential therapeutic agents for severe COVID-19, Hu et al. from the US. reported the suppression of production of multiple cytokines by 17α-hydroxyprogesterone caproate (17-OHPC), based on their results from *in vitro* cytokine release assay using human peripheral blood mononuclear cells (PBMCs) and *in vivo* assay using PBMC-engrafted immunodeficient mice stimulated with OKT3. 17-OHPC is a synthetic progestogen that exhibits potent anti-inflammatory and immuno-modulatory activities, at least partially, by agonism of progesterone receptor and selective modulation of glucocorticoid receptor and subsequent regulation of downstream pathways, such as NF-κB signaling (Gerber et al., 2009). This compound was also shown to prevent airway remodeling and pulmonary fibrosis in animal studies (Zhang et al., 2018), with potential to provide additional benefits for COVID-19 patients. The phase II clinical trial of 17-OHPC is currently in progress, and its potential use is also partially supported by the fact that male gender is a risk factor for COVID-19 severity and mortality (Jin et al., 2020).

In another effort of developing new therapies for COVID-19, Takenaka et al. from Japan compared the anti-inflammatory effects of five FDA-approved selective serotonin reuptake inhibitors (SSRIs) including paroxetine, fluoxetine, fluvoxamine, sertraline and escitalopram, in terms of the inhibition on IL-6 production in various immune cells upon different stimuli. Fluoxetine was identified as the most promising candidate as an anti-inflammatory drug to mitigate hyper-inflammatory responses and to treat cytokine release syndrome, given its potent efficacy and low cytotoxicity among five SSRIs. In fact, the potential application of SSRIs, particularly fluoxetine in the treatment of COVID-19 was also supported by clinical observations. In a multicenter cohort study analyzing electronic health records of 83,584 COVID-19 patients, 8 and 28% reductions in the relative risk of mortality were found among patients prescribed any SSRI and in those prescribed fluoxetine, respectively (Oskotsky et al., 2021).

In summary, this Research Topic is a collection of meta-analysis, review and original researches related to cytokine release syndrome in COVID-19 and possible therapeutic strategies. These articles are valuable resources to advance our current understandings of COVID-19 pathogenesis and therapies. In addition to viral infection, of note, cytokine release syndrome is also associated with graft-vs.-host disease, hemophagocytic lymphohistiocytosis, sepsis, antibody therapy, and chimeric antigen receptor (CAR)-T cell therapy, etc (Karki and Kanneganti, 2021). In this regard, the knowledge presented in this Research Topic also has the potential to be applied to much broader therapeutic areas in the future.
